# [Expression of Concern] Antitumor activity of Notch-1 inhibition in human colorectal carcinoma cells

**DOI:** 10.3892/or.2026.9146

**Published:** 2026-06-02

**Authors:** Wangdi Liao, Guohua Li, Yu You, Hongping Wan, Qiong Wu, Chongwen Wang, Nonghua Lv

Oncol Rep 39: 1063–1071, 2018; DOI: 10.3892/or.2017.6176

Following the publication of this paper, it was drawn to the Editor's attention by a concerned reader that, regarding the cell morphology experiments shown in Fig. 4C and 9B on p. 1066 and p. 1069 respectively, the ‘NC’ data panel in Fig. 4C appeared to match with the data shown to represent the ‘Control’ data panel in Fig. 9B, albeit the images appeared in these figures at different sizes.

The authors have been contacted by the Editorial Office to offer an explanation for the apparent re-use of the same data in this paper, and we are awaiting their response. Owing to the fact that the Editorial Office has been made aware of potential issues surrounding the scientific integrity of this paper, we are issuing an Expression of Concern to notify readers of this potential problem while the Editorial Office continues to investigate this matter further.

